# Micro‐ultrasound tissue echogenicity predicts prostate cancer grade

**DOI:** 10.1002/bco2.70192

**Published:** 2026-04-13

**Authors:** Ailene E. Corona, Dan Luca, Kian Kolahi Sohrabi, Kyla Grunden, William Chen, Pooneh J. Sarmadian, Kevin J. Walsh, Lorna Kwan, Qi Miao, Kyung Sung, Wayne G. Brisbane

**Affiliations:** ^1^ Department of Urology University of California Los Angeles California USA; ^2^ Department of Radiology University of California Los Angeles California USA

**Keywords:** echogenicity, micro‐ultrasound, prostate cancer, prostate imaging, ultrasound

## Abstract

**Objectives:**

This study aimed to evaluate the association between lesion echogenicity on micro‐ultrasound (micro‐US) and the presence and grade of prostate cancer.

**Patients and Methods:**

We prospectively analysed 229 prostate lesions from 181 men undergoing micro‐US‐guided transperineal biopsy at UCLA. Lesions were visually graded as hyperechoic, isoechoic or hypoechoic relative to the central zone, which served as an internal reference due to its consistent tissue characteristics and low malignancy risk. Biopsy targeting was confirmed by visualizing the needle tract within the lesion. The primary outcome was the detection rate of Grade Group (GG) ≥ 2 cancer across echogenicity categories. Secondary analyses included associations with PSA density and MRI‐derived apparent diffusion coefficient (ADC) values, given that ADC is associated with tissue cellularity. Statistical comparisons were performed using chi‐square and Kruskal–Wallis tests, with post hoc pairwise analyses where appropriate.

**Results:**

GG ≥ 2 cancer detection rates increased with decreasing echogenicity: 22% in hyperechoic, 56.2% in isoechoic and 62.4% in hypoechoic lesions (*p* < 0.01). Hypoechoic lesions also exhibited a higher proportion of GG ≥ 3 cancers (*p* < 0.05). ADC values declined progressively from hyperechoic to hypoechoic lesions (median: 980, 851 and 751, respectively; *p* < 0.01), suggesting higher tissue cellularity. Regression analysis demonstrated no meaningful interaction between PRI‐MUS and echogenicity. PSA density did not significantly differ among echogenicity groups.

**Conclusions:**

Lesion hypoechogenicity on micro‐US is strongly associated with higher grade prostate cancer and lower ADC values, suggesting a link to tissue cellularity. These findings support the incorporation of echogenicity as a diagnostic marker within the micro‐US PRI‐MUS framework, potentially enhancing the accuracy of prostate cancer risk stratification.

## 1 INTRODUCTION

Prostate cancer is the most common malignancy in men and a leading cause of cancer‐related death worldwide.^1^, ^2^ Imaging plays a key role in the detection and characterization of prostate cancer. Since around 2010, multiparametric magnetic resonance imaging (MRI) has been used to localize suspicious regions and guide targeted biopsy. More recently, MRI has been compared to a novel high‐resolution ultrasound platform—micro‐ultrasound (Micro‐US), which uses high‐frequency (29 MHz) sound waves to achieve anatomical resolution down to 70 μm.^3^ The OPTIMUM trial, an international, 20‐centre study involving over 800 patients, found that micro‐US and MRI had equivalent prostate cancer detection rates.^4^

Although both modalities detect cancer, their underlying physics differ. In MRI, a key marker is the apparent diffusion coefficient (ADC), which reflects tissue density. Prostate cancers exhibit low ADC values due to restricted water molecule movement in densely packed tumour cells.^5^, ^6^ In contrast, micro‐US relies on differences in reflected sound waves. Benign and cancerous tissues reflect ultrasound waves with varying intensities: Dense cancer tissue attenuates incident sound waves away from the transducer, and irregular tumour borders scatter echoes, resulting in decreased signal intensity.^7^ This hypoechoic appearance is consistent with conventional ultrasound (5–7 MHz), where prostate cancer appears hypoechoic in 60%–80% of cases.^8^, ^9^, ^10^ However, the hypoechoic effects of cancer are likely amplified at higher frequencies, and micro‐US may be more sensitive to internal fibrotic or cellular tumour tissues, further lowering echo intensity.

The micro‐US scoring system, Prostate Risk Identification Using Micro‐Ultrasound (PRI‐MUS), disproportionately represents hypoechoic patterns among the acoustic patterns classified as PRI‐MUS 4 and 5. However, PRI‐MUS does not incorporate a quantitative echogenicity evaluation analogous to the ADC in MRI. For the MRI‐based PI‐RADS system, a lesion must demonstrate restricted diffusion to be quantified as a PI‐RADS 4 or 5 lesions. Within PRI‐MUS 4 lesions, both the ‘starry sky and “cauliflower”’ patterns are hyperechoic.

In this study, we evaluated lesion echogenicity on micro‐US relative to an internal fiducial (central zone) and quantified its association with cancer risk at the time of biopsy. As a secondary objective, we examined the correlation between micro‐US lesion echogenicity and MRI ADC values to assess whether echogenicity reflects underlying tissue density.

## 2 METHODS

This study included men undergoing micro‐US guided prostate biopsy at the University of California, Los Angeles Medical Center from January 2021 through March 2025. Men presenting for biopsy obtained a pre‐biopsy MRI utilizing a 3‐T scanner. A list of MRI characteristics is available in Table S1. Men were considered eligible for biopsy if there was a lesion on MRI (PI‐RADS 3–5), or when an MRI was negative, but PSA density was greater than 0.15 ng/mL. All men undergoing biopsy were prospectively entered into a database maintained by a dedicated research coordinator (co‐author KG).

For this study, we evaluated a cohort of 181 sequential biopsy patients. MRI lesions were targeted on Micro‐US utilizing anatomic fiducial based cognitive fusion.^11^ First, the MRI ROI was isolated into a prostate region (i.e., right posterior apex). We then noted anatomic fiducials adjacent to the ROI (e.g., verumontanum, interface of the transition and peripheral zone or visible cysts). At the time of the micro‐ultrasound guided biopsy, we visualized the MRI fiducials on micro‐US and then refined our biopsy locations using the cancer characteristics characterized by PRI‐MUS. If no PRI‐MUS lesion was visible, we placed three cores in the direct vicinity of the MRI anatomic fiducial. We have previously demonstrated that this anatomic fiducial‐based cognitive fusion approach has a cancer detection rate of up to 97%.^12^

All cognitive registration and subsequent biopsies were performed by a fellowship‐trained urologist with more than 500 cases of experience in Micro‐US imaging and Level 4 mastery as described in the OPTIMUM clinical trial (co‐author WB).^4^ If there were additional Micro‐US regions of interest not visible on MRI, these were also biopsied. We sampled each imaging lesion (MRI, Micro‐US or dual imaging visible) with three targeted cores. Systematic cores were added for patients with equivocal ROI visibility on Micro‐US but omitted for dual imaging visible MRI and micro‐ultrasound targets (PI‐RADS + PRI‐MUS ≥ 4). As previously mentioned, this approach reduces the need for systematic biopsy while maintaining a 97% cancer detection rate.^12^

**FIGURE 1:**
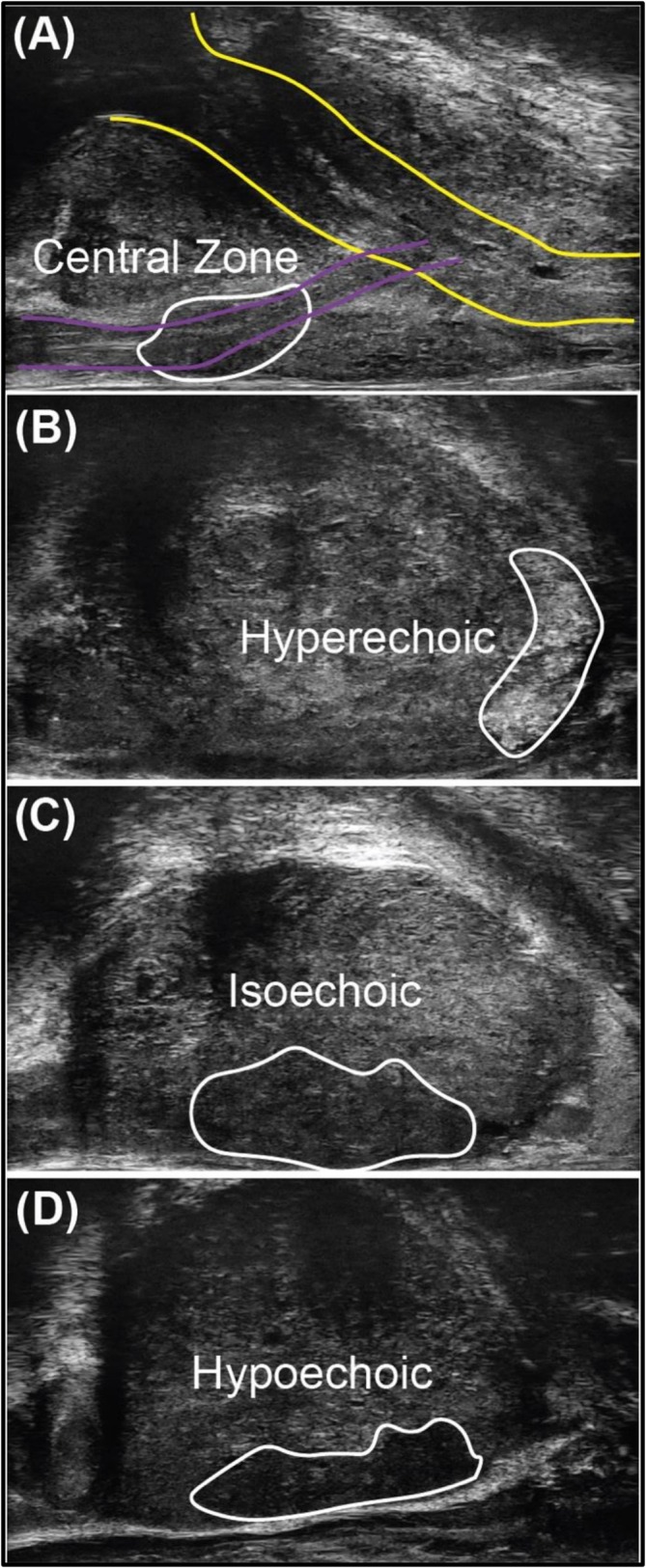
Prostate cancer lesions on micro‐ultrasound exhibiting varying echogenic properties. By default, micro‐US is visualized in the sagittal plane, with the bladder (proximal) on the left of the image, penis (distal) on the right of the image, pubis (anterior) at the top of the image and rectum (posterior) at the bottom of the image. All lesions were biopsied with three target cores. (A) Micro‐ultrasound image with the urethra (yellow), ejaculatory ducts (purple) and central zone (white) displayed in the sagittal plane. (B–D) Micro‐ultrasound lesions with (B) hypoechoic, (C) isoechoic and (D) hypoechoic lesions (white outline).

Each Micro‐US region of interest we assigned a PRI‐MUS score based on the acoustic features described by Ghai et al.^13^ The PRI‐MUS score utilizes visual acoustic patterns such as ‘Swiss cheese’, ‘cauliflower’ and ‘starry sky’. However, in addition, we evaluated the Micro‐US tissue echogenicity and scored it compared to the central zone of the prostate. The central zone was chosen given it represents cellularly dense glandular tissue. The central zone contains small ducts analogous to Gleason 3+3 prostate cancer; however, it rarely gives rise to primary prostate cancer.^14^ By contrast, prostate cancer within the peripheral zone, the location of 80% of prostate cancers, represents the transformation of large benign glands to consolidated cancerous glands.^15^ The naturally small glandular structure of the central zone enables a convenient internal reference with some similarities to low‐grade cancer, but minimal risk of missing a primary prostate malignancy. Micro‐US regions of interest were categorized compared to central zone echogenicity as (1) hyperechoic—brighter than the central zone, (2) isoechoic—equivalent brightness to the central zone and (3) hypoechoic—darker than the central zone (Figure 1).

**FIGURE 2:**
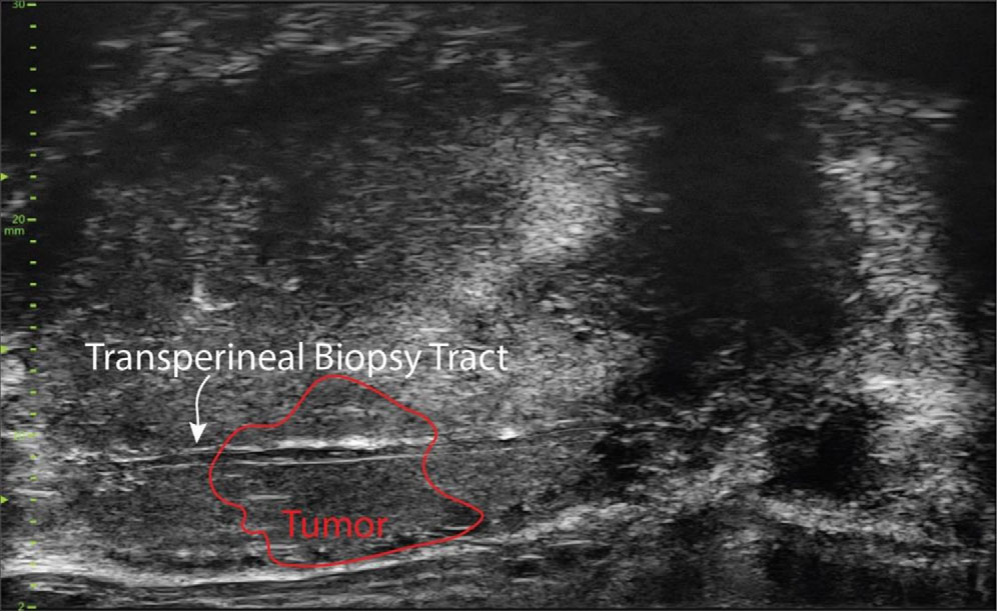
Micro‐ultrasound image with a biopsy tract following tissue removal with an 18‐gauge biopsy needle. Imaging parameters of all tissue samples were recorded via a cine sweep of the post‐biopsy tissue. For example, this target bounded in red was considered isoechoic.

We ensured that the targeted biopsy had traversed the Micro‐US region of interest by visualizing the 18‐gauge biopsy needle cavity through the region of interest (Figure 2). This cavity is not visible on conventional ultrasound but is visualized with the increased resolution of Micro‐US. By grading the echogenicity and PRI‐MUS of each Micro‐US region of interest, and visually confirming the biopsy of the same ROI, we compared the grade group, PRI‐MUS score and tissue echogenicity. As the Micro‐US ROI was also targeted from an MRI ROI, we also performed an exploratory analysis of the PI‐RADS score and ADC with echogenicity. ADC analysis was performed on a subset of lesions (*n* = 100). These lesions were MRI and Micro‐US concordant.

### 2.1 Outcomes

Our primary outcome of interest was the association between lesion echogenicity and the presence of Grade Group (GG) ≥ 2 prostate cancer. We assigned the imaging target Grade Group from the highest grade of the three targeted cores. We calculated the GG ≥ 2 cancer detection rate for each echogenicity category and analysed the grade group distribution across the three echogenicity categories to determine whether echogenicity was associated with grade severity. We also calculated the GG ≥ 2 cancer detection rate for each PRI‐MUS score and evaluated the distribution of echogenicity across PRI‐MUS scores (3–5) among lesions with clinically significant prostate cancer. In addition, we conducted a logistic regression analysis including PRI‐MUS classification and echogenicity, as well as their interaction term, to evaluate whether echogenicity provides additional predictive value beyond PRI‐MUS.

Furthermore, a multivariable logistic regression analysis was performed with the presence of GG ≥ 2 prostate cancer as the outcome, including age, prostate‐specific antigen density (PSAD) and echogenicity as covariates, to assess the independent predictive value of echogenicity after adjustment for the other variables. Finally, lesions were stratified by anatomical location, and the association between echogenicity and GG ≥ 2 prostate cancer was evaluated within each region (anterior prostate, posterior prostate and lesions spanning both regions).

In a secondary analysis, we evaluated the association of lesion echogenicity with prostate‐specific antigen levels, PSA density and MRI‐determined ADC. We were interested in the correlation of echogenicity and ADC, given that lower ADC is correlated with increased tissue cellularity.^6^, ^16^ We hypothesized that echogenicity may be an acoustic marker for cell density.

### 2.2 Statistical analysis

The association between lesion echogenicity and the presence of GG ≥ 2 cancer was evaluated using Pearson's chi‐squared test. Post hoc pairwise chi‐squared tests were performed to identify significant differences between echogenicity categories. Multiple comparison corrections (Bonferroni and Holm) were applied to control for Type I error inflation. ADC values were analysed using the Kruskal–Walli's rank sum test to compare differences across the three echogenicity groups. Dunn's pairwise comparisons were then conducted to assess statistical significance between median ADC values for each echogenicity category. A *p*‐value < 0.05 was considered statistically significant.

## 3 RESULTS

Our study included 181 men with a mean age of 69 years. A total of 229 lesions were analysed, comprised of 181 primary/index lesions, 46 secondary lesions and two tertiary lesions. Patient features were consistent with expected parameters for prostate cancer, including a mean age of 69 years and a median PSA of 7.32. Our population was around 65% Caucasian (Table 1).

**TABLE 1:** Baseline demographics and characteristics.

Variable	Total patient (*N* = 181)
Age (years) and mean (SD)	69 (8.4)
Ethnicity	
African American	15 (8.2%)
American Indian	3 (1.6%)
Asian	17 (9.3%)
Caucasian	118 (64.8%)
Hispanic	8 (4.4%)
Choose not to disclose	20 (11%)
PSA (ng/mL), median (IQR)	7.32 (6.05)
Micro‐Ultrasound PSAD (ng/mL^2^), median (IQR)	0.215 (0.23)
Total lesions	229
Primary	181 (79%)
Secondary	46 (20.1%)
Tertiary	2 (0.9%)

**FIGURE 3:**
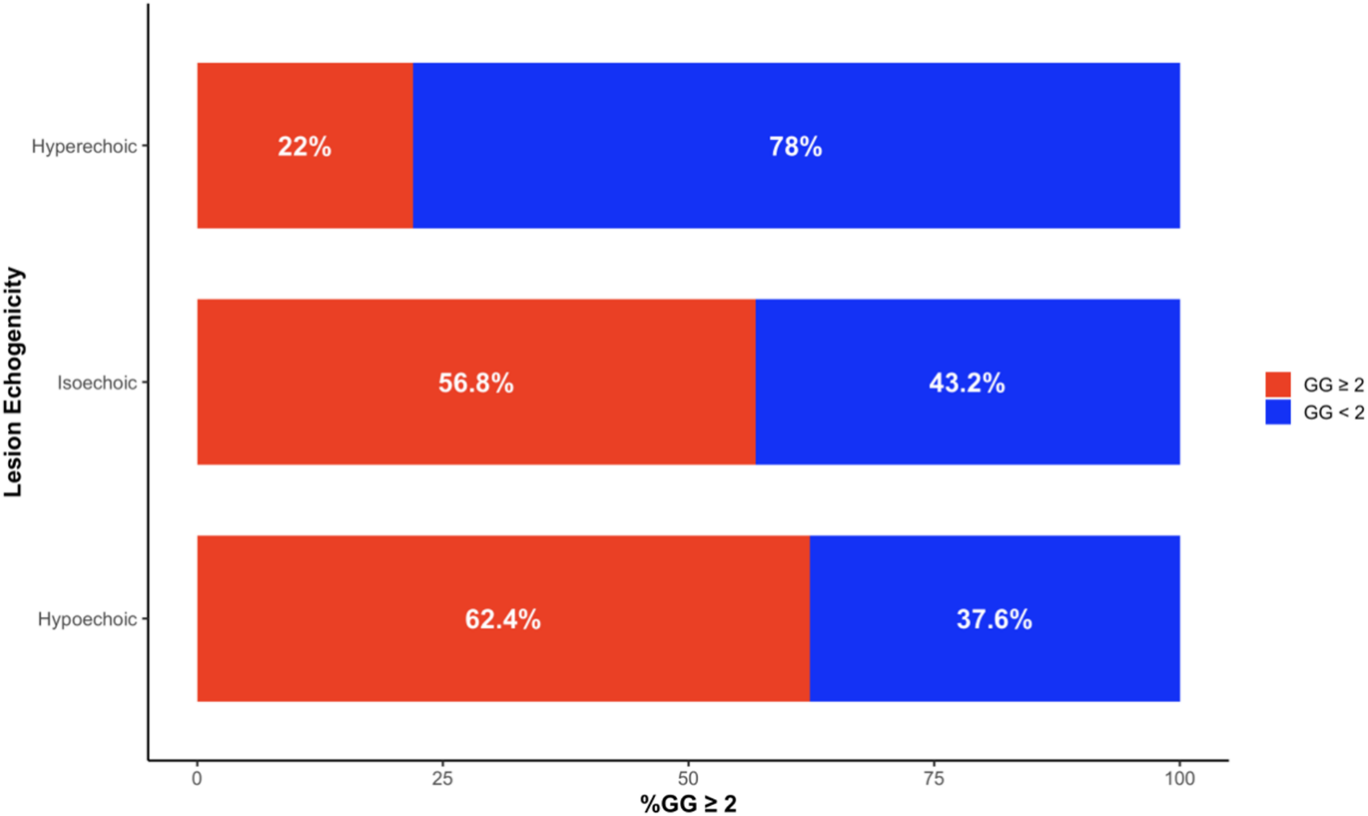
Percentage of grade group ≥2 detection by echogenicity levels (*p* < 0.05). Post hoc pairwise chi‐square tests: hyperechoic vs. isoechoic (*p* < 0.01), hyperechoic vs. hypoechoic (*p* < 0.01) and isoechoic vs. hypoechoic (*p* > 0.05).

The incidence of GG ≥ 2 cancer significantly increased as lesion echogenicity darkened from hyperechoic to hypoechoic (Figure 3, *p* < 0.05). Hyperechoic lesions exhibited 22% GG ≥ 2, compared to 56.2% isoechoic and 62.4% hypoechoic lesions (Figure 3). Pairwise analysis demonstrated a significant difference between hyperechoic and isoechoic lesions and between hypoechoic and hyperechoic lesions (*p* < 0.01). The difference between the proportion of GG2 cancers in isoechoic versus hypoechoic lesions did not reach statistical significance (*p* > 0.05). Furthermore, there was no significant difference in csPCa detection between anterior and posterior lesions across echogenicity categories. However, we observed that lesions spanning both anterior and posterior regions demonstrated a markedly higher proportion of csPCa, particularly among hypoechoic (78%) and isoechoic (69%) lesions (Figure S1).

**FIGURE 4:**
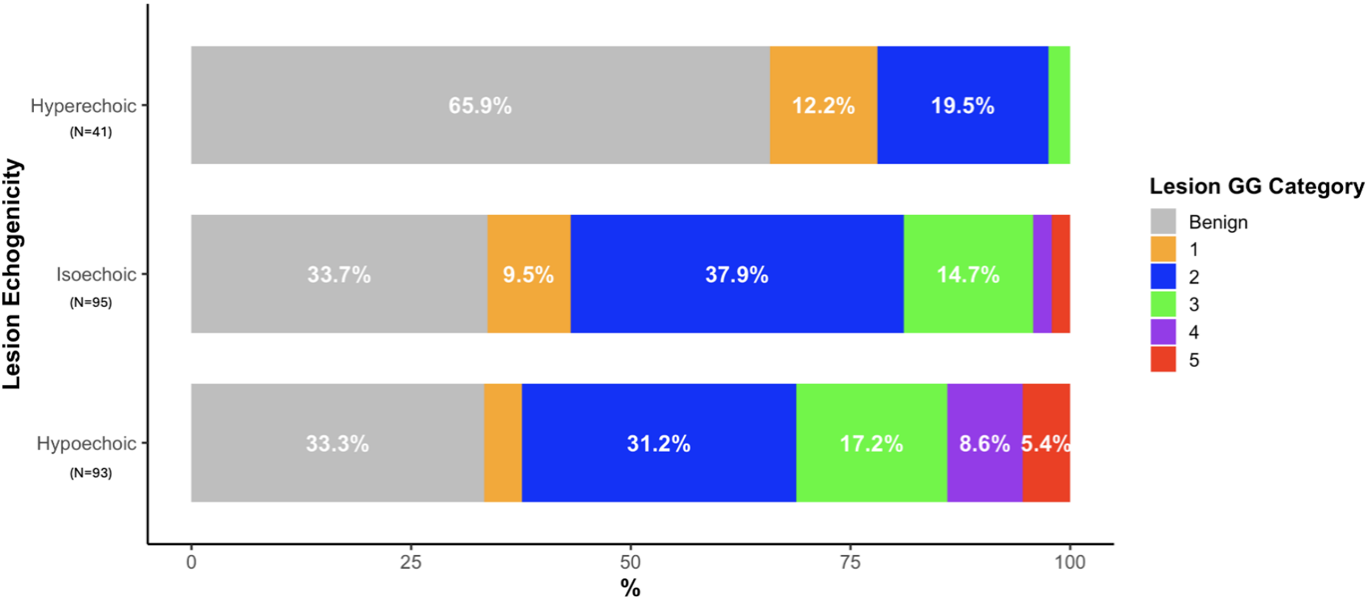
Distribution of lesion GG across lesion echogenicity categories. A chi‐square test for independence showed a statistically significant difference in distribution (*χ* ^2^ = 29.46, df = 10, *p* < 0.01).

Analysis of Grade Group (benign and GG 1–5) distribution also demonstrated a significant difference across each echogenicity category (*p* < 0.01, Figure 4). Hyperechoic lesions tended to include a higher proportion of benign tissue (66%) compared to around 33% for isoechoic and hypoechoic lesions. A subgroup analysis evaluating the proportions of GG 2–5 for each echogenicity category demonstrated that hypoechoic and isoechoic lesions had lower proportions of GG2 compared to hyperechoic lesions (50% and 67% vs. 89%). These differences did not reach significance (*p* = 0.22); however, GG 4 and 5 tumours were absent in the hyperechoic category, while increasingly prevalent for isoechoic (7%) and hypoechoic lesions (22%).

**FIGURE 5:**
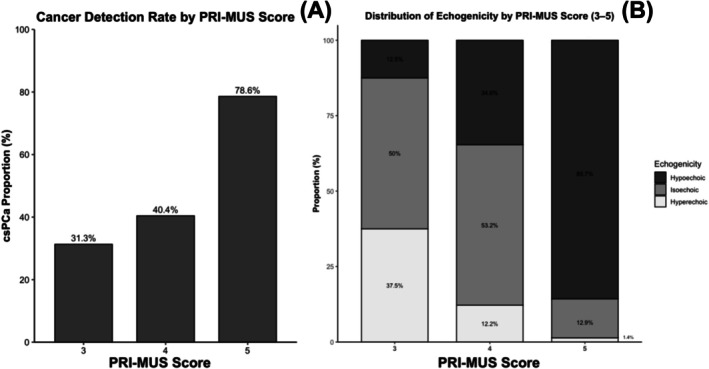
PRI‐MUS cancer detection rate and echogenicity distribution. (A) Cancer detection rate for clinically significant prostate cancer (csPCa; GG ≥ 2) stratified by PRI‐MUS score (3–5). Values represent proportions expressed as percentages. B. Distribution of echogenicity by PRI‐MUS score (3–5) among lesions with GG ≥ 2 prostate cancer. Stacked bars represent the proportional distribution of hyperechoic, isoechoic and hypoechoic lesions within each PRI‐MUS score category.

In our cohort, the detection rate of clinically significant prostate cancer (GG ≥ 2) increased across PRI‐MUS scores (Figure 5A). The csPCa detection rate was 31.3% for PRI‐MUS 3 lesions, 40.4% for PRI‐MUS 4 lesions and 78.6% for PRI‐MUS 5 lesions. Among lesions with GG ≥ 2 prostate cancer, the distribution of echogenicity across PRI‐MUS scores is shown in Figure 5B. Hypoechoic lesions accounted for 12.5% of PRI‐MUS 3 lesions, 34.6% of PRI‐MUS 4 lesions and 85.7% of PRI‐MUS 5 lesions. Correspondingly, the proportion of hyperechoic lesions decreased from 37.5% in PRI‐MUS 3 to 12.2% in PRI‐MUS 4 and 1.4% in PRI‐MUS 5 lesions. Logistic regression analysis including PRI‐MUS classification, echogenicity, and their interaction term demonstrated no meaningful interaction between PRI‐MUS and echogenicity. Echogenicity demonstrated an independent main effect, with hyperechoic lesions being significantly less likely to harbour csPCa compared with isoechoic lesions (*p* = 0.025), even after adjustment for PRI‐MUS classification.

In multivariable logistic regression analysis adjusting for age and PSAD, hyperechoic lesions were significantly less likely to harbour clinically significant prostate cancer (csPCa; GG ≥ 2) compared with isoechoic lesions (adjusted OR 0.30, 95% CI 0.13–0.66; *p* = 0.003). Hypoechoic lesions demonstrated higher odds of csPCa relative to isoechoic lesions; however, this association did not reach statistical significance (adjusted OR 1.22, 95% CI 0.73–2.06; *p* = 0.440). Full multivariable regression results are provided in Figure S2.

**FIGURE 6:**
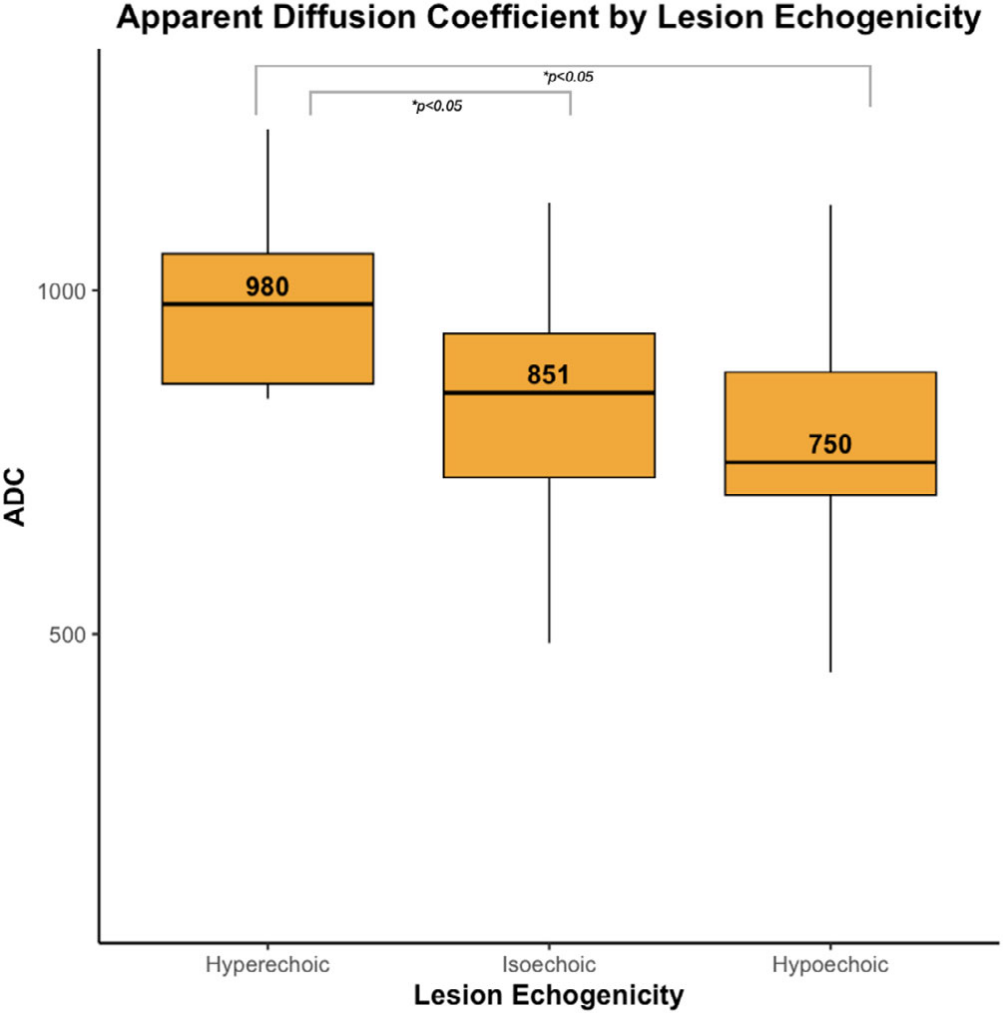
Lesion ADC values across echogenicity levels. Statistical comparisons were performed using the Kruskal–Wallis test, followed by Dunn's post hoc test for pairwise comparisons.

When evaluating the ADC values for each category of lesion echogenicity, there was a decrease in ADC (lower = more cellular) from hyperechoic to isoechoic to hypoechoic, indicating an increased cellular density with decreased echogenicity (Figure 6). The difference in ADC between echogenicity categories was significant (*p* < 0.01). We also evaluated PSA density across echogenicity; no statistically significant differences were observed in median PSA density across the three groups.

## 4 DISCUSSION

In this study, we demonstrate a strong association between micro‐US tissue echogenicity and prostate cancer grade. Hypoechoic lesions were significantly more likely to contain GG ≥ 2 prostate cancer, and we noted a stepwise increase in cancer prevalence and grade severity as echogenicity decreased. Echogenicity also correlated with ADC measured on MRI. Supporting the hypothesis that decreased echogenicity reflects higher tissue cellularity, a hallmark of aggressive disease. As the first study, to our knowledge, to systematically characterize echogenicity on Micro‐US, our findings are intended to be hypothesis‐generating and to inform future research aimed at refining the diagnostic utility of echogenicity and its potential integration into established frameworks such as PRI‐MUS.

Our findings reaffirm and build upon prior literature recognizing that prostate cancer is often hypoechoic on ultrasound.^8^, ^9^ However, while conventional ultrasound is unable to differentiate benign and cancerous tissue, the OPTIMUM trial demonstrated micro‐US identifies cancer with similar accuracy to MRI.^4^ The micro‐US grading system, PRI‐MUS, batches acoustic patterns by their risk of GG ≥ 2 cancer.^13^ PRI‐MUS 5 lesions are all isoechoic and hypoechoic. Conversely, PRI‐MUS 3 lesions are all hyperechoic. However, PRI‐MUS 4 lesions contain both patterns, ranging from brightly hypoechoic to hypoechoic. Unsurprisingly, the cancer detection rate for PRI‐MUS 4 lesions varies.^4^, ^12^, ^17^

We fully acknowledge that the PRI‐MUS protocol is a validated scoring system that incorporates echogenicity and additional acoustic features into its classification framework. In our dataset, a strong relationship between PRI‐MUS classification and echogenicity was observed, with the proportion of hypoechoic lesions increasing across higher PRI‐MUS scores, reflecting an expected correlation between these features. Consistent with this overlap, regression analysis demonstrated no meaningful interaction between PRI‐MUS and echogenicity, indicating that echogenicity does not provide additional predictive value beyond the PRI‐MUS score itself.

Nevertheless, echogenicity demonstrated an independent main effect, with hyperechoic lesions being significantly less likely to harbour clinically significant prostate cancer compared with isoechoic lesions, even after adjustment for PRI‐MUS classification. While we acknowledge that proposing echogenicity as a novel standalone biomarker for prostate cancer would be premature, we believe these findings remain clinically relevant. For example, among the 37 lesions categorized as PRI‐MUS 3 or 4 that were hyperechoic, only 16% contained clinically significant prostate cancer, suggesting that echogenicity may provide useful contextual information when interpreting equivocal lesions.

We could improve the PRI‐MUS system by restricting four and five lesions to those with isoechoic or hypoechoic patterns and moving all hyperechoic lesions to PRI‐MUS 3. In our cohort, hypoechoic lesions were significantly more likely to harbour GG ≥ 2 cancer compared to hyperechoic lesions (62.4% vs. 22%). Additionally, hypoechoic lesions contained more aggressive grade cancers and correlated with increased cellular density as measured by MRI‐based ADC.

Our study details the relationship between micro‐US tissue echogenicity and prostate cancer, but several limitations should be considered. First, this is a single‐centre, single‐surgeon analysis. While the data were prospectively collected, a larger multi‐institutional study is needed to assess inter‐reader variability and the learning curve across different experience levels. Second, echogenicity was classified qualitatively, whereas a quantitative measurement, similar to MRI‐based ADC could reduce variability. We feel the central zone provides a convenient internal reference, with a low incidence of primary malignancy; however, this zone has not previously been used as a reference. The lack of precedence is due to the unique resolution of micro‐US that differentiates the central zone from the adjacent peripheral zone. Finally, we based our ground truth cancer designation on a biopsy cohort rather than surgical specimens. A benefit of a surgical cohort is the ability to evaluate all regions within the prostate and identify very small cancers usually missed on imaging. However, we elected for a biopsy‐based evaluation for three reasons. First, micro‐US is able to visualize needle passes through the ROI (Figure 3), ensuring that the ROI is sampled. Second, surgical cohorts lack benign tissue. While there are benign areas within a cancer‐afflicted prostate, cancer likely causes a whole gland phenotype change, influencing tissue echogenicity.^18^ Prior evaluation of MRI using surgical and biopsy cohorts produces markedly different results.^19^, ^20^ Because PRI‐MUS will function for biopsy risk stratification, a biopsy‐based cohort is most appropriate. However, we note that the correlation of echogenicity and ADC is only an association. While we prospectively register Micro‐US and MRI lesions prior to biopsy, we can only confidently state that micro‐US guided biopsy core samples the exact tissue from the same general region measured by MRI ADC. Future work could directly measure tissue cellularity and echogenicity and include genomics and hypoxia as other causes of micro‐US tissue appearance.

## 5 CONCLUSION

This study demonstrates that hypoechoic lesions on Micro‐US contain more cancer of higher grades. We observed a stepwise increase in the proportion of GG ≥ 2 cancer with darkening echogenicity. The relationship between ADC values and cancer echogenicity further supports the utility of echogenicity as a diagnostic marker. An interesting future direction includes a multi‐reader study and incorporating echogenicity into the micro‐US PRI‐MUS grading system.

## AUTHOR CONTRIBUTIONS

**Ailene E. Corona:** data curation, investigation project administration. **Dan Luca:** conceptualization, investigation, formal analysis, supervision, writing – original draft preparation. **Kian Kolahi Sohrabi:** data curation, methodology. **Kyla Grunden:** data curation, resources. **William Chen:** investigation, data curation. **Pooneh J. Sarmadian:** data curation. **Kevin J. Walsh:** investigation, validation. **Lorna Kwan:** methodology, data curation, formal analysis. **Qi Miao:** investigation, writing – review and editing. **Kyung Sung:** investigation, writing – review and editing. **Wayne G. Brisbane:** supervision, conceptualization, funding acquisition, investigation, writing – review and editing.

## CONFLICT OF INTEREST STATEMENT

Dr. Brisbane is a consultant for Exact Imaging—manufacturer of the Micro‐Ultrasound machine. None of these companies sponsored this research. All other authors have no conflicts of interest or disclosures to report.

## Supporting information


**Figure S1:** Proportion of clinically significant prostate cancer stratified by lesion location and echogenicity.
**Figure S2.** Multivariable logistic regression model evaluating the association between lesion echogenicity and clinically significant prostate cancer. The outcome was the presence of **Grade Group ≥ 2 prostate cancer**. Echogenicity was modeled as a categorical variable with **isoechoic lesions serving as the reference category**. The model was adjusted for **age** and **PSA density (PSAD),** and results are reported as adjusted odds ratios with 95% confidence intervals.

**Table S1.** MRI Characteristics for Magnetom Skyra 3 T Scanner used at UCLA.
