# Supplementary material for: Micro‐ultrasound tissue echogenicity predicts prostate cancer grade
Source: BJUI Compass. 2026 Apr 13;7(4):e70192. doi: 10.1002/bco2.70192 (PMC13077212; doi:10.1002/bco2.70192)
Supplement: Supplementary file 1 — Figure S1: Proportion of clinically significant prostate cancer stratified by lesion location and echogenicity. Figure S2. Multivariable logistic regression model evaluating the association between lesion echogenicity and clinically significant prostate cancer. The outcome was the presence of Grade Group ≥ 2 prostate cancer. Echogenicity was modeled as a categorical variable with isoechoic lesions serving as the reference category. The model was adjusted for age and PSA density (PSAD), and results are reported as adjusted odds ratios with 95% confidence intervals. [file BCO2-7-e70192-s002.docx]

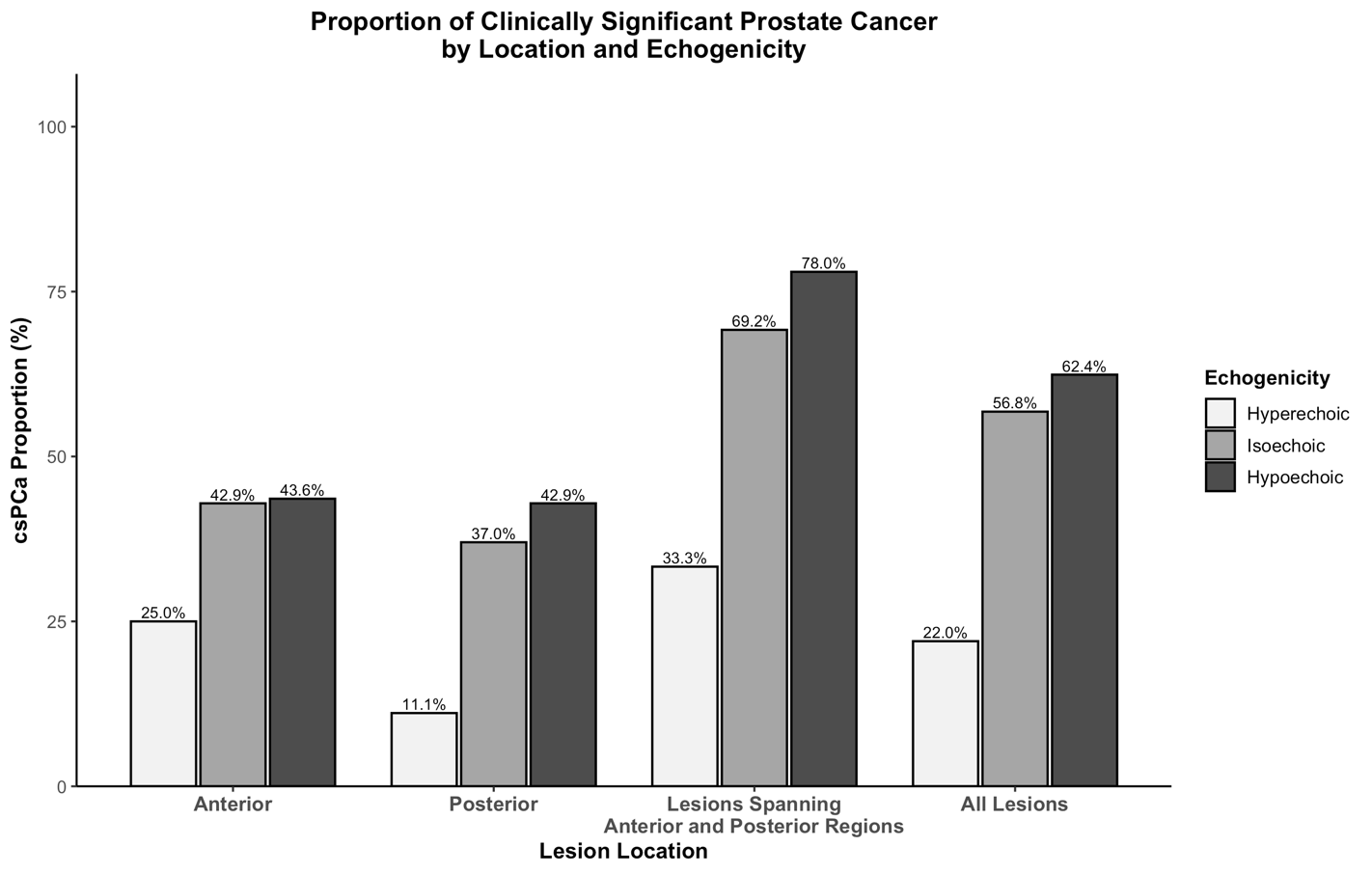


**Supplemental Figure 1:** Proportion of clinically significant prostate cancer stratified by lesion location and echogenicity.

*
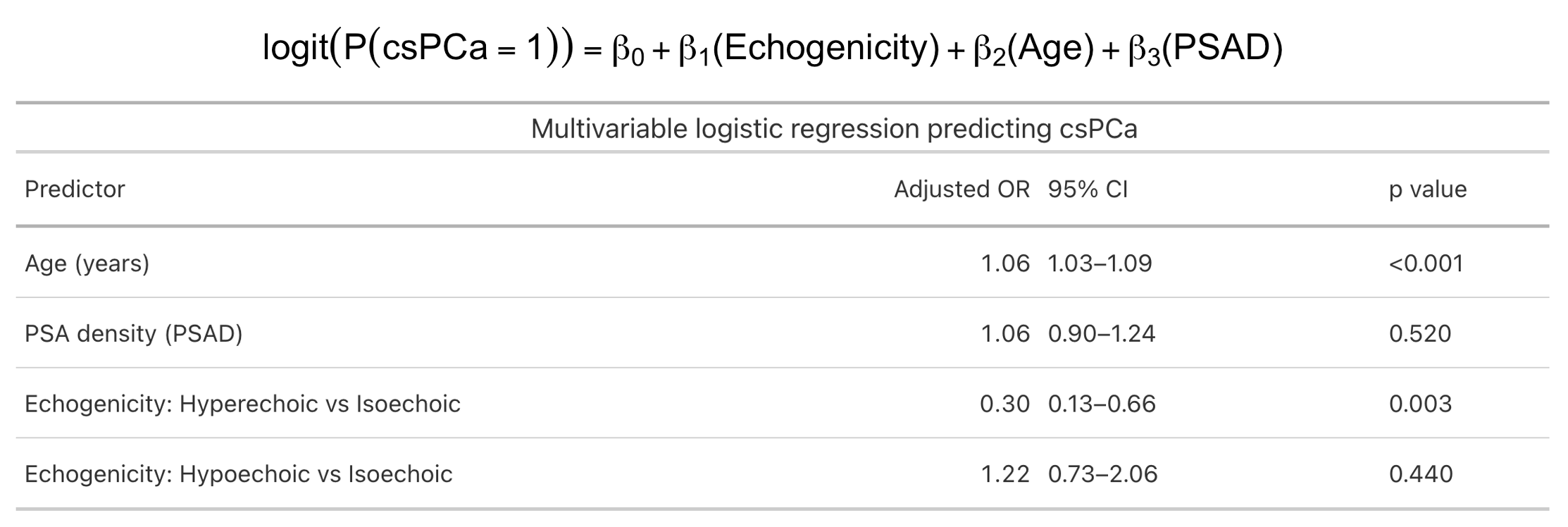
*

**Supplementary Figure 2.** Multivariable logistic regression model evaluating the association between lesion echogenicity and clinically significant prostate cancer. The outcome was the presence of **Grade Group ≥ 2 prostate cancer**. Echogenicity was modeled as a categorical variable with **isoechoic lesions serving as the reference category**. The model was adjusted for **age** and **PSA density (PSAD),** and results are reported as adjusted odds ratios with 95% confidence intervals.
