# Supplementary figures and images for: Micro‐ultrasound tissue echogenicity predicts prostate cancer grade
Source: BJUI Compass. 2026 Apr 13;7(4):e70192. doi: 10.1002/bco2.70192 (PMC13077212; doi:10.1002/bco2.70192)

**
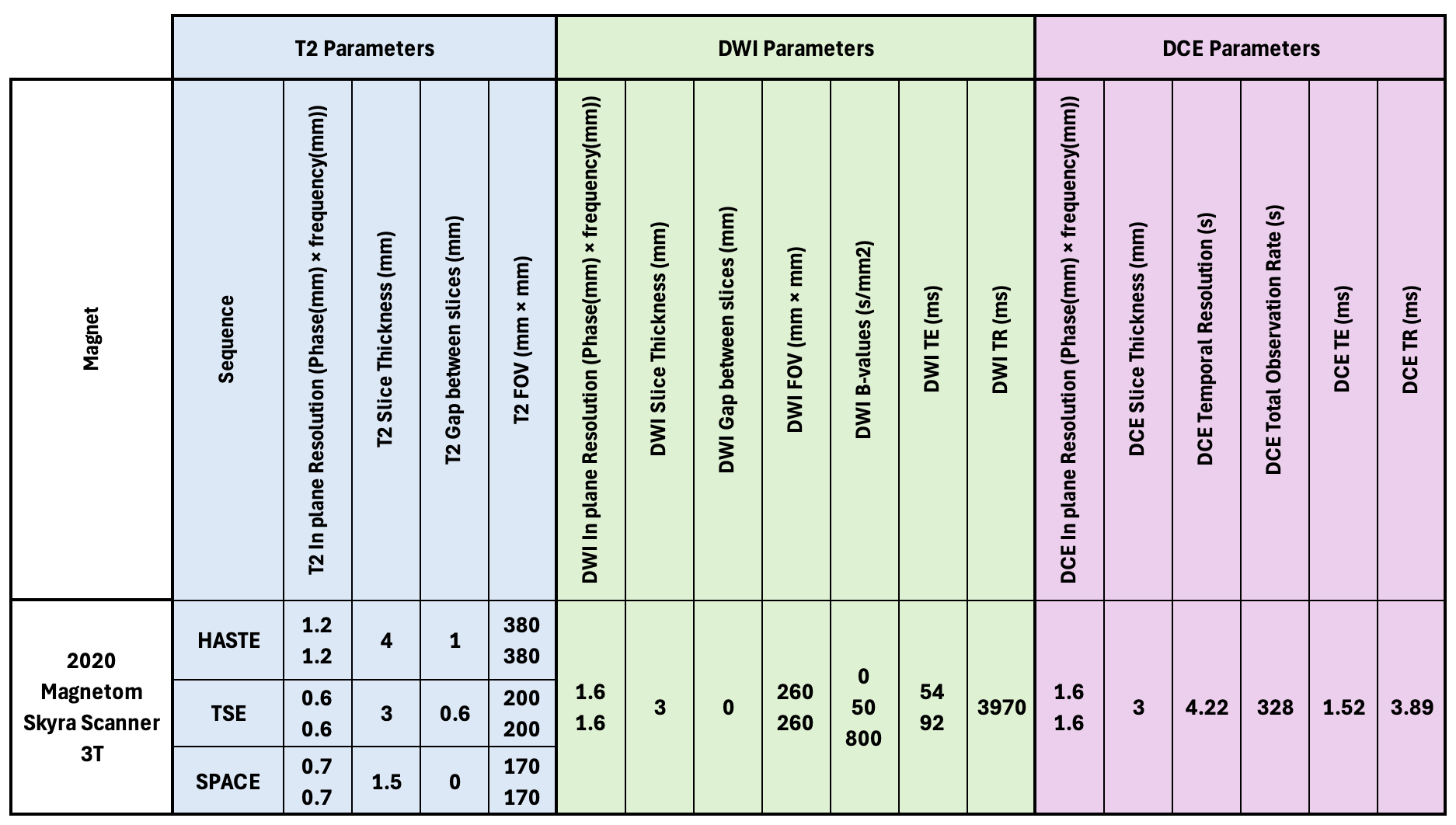
**

**Supplemental Table 1.** MRI Characteristics for Magnetom Skyra 3T Scanner used at UCLA.

Supplement: Supplementary file 2 — Table S1. MRI Characteristics for Magnetom Skyra 3 T Scanner used at UCLA. [file BCO2-7-e70192-s001.docx]
